# Data consistency in the English Hospital Episodes Statistics database

**DOI:** 10.1136/bmjhci-2022-100633

**Published:** 2022-10-28

**Authors:** Flavien Hardy, Johannes Heyl, Katie Tucker, Adrian Hopper, Maria J Marchã, Tim W R Briggs, Jeremy Yates, Jamie Day, Andrew Wheeler, Sue Eve-Jones, William K Gray

**Affiliations:** 1 Getting It Right First Time, NHS England and NHS Improvement London, London, UK; 2 Department of Physics and Astronomy, University College London, London, UK; 3 Innovation and Intelligent Automation Unit, Royal Free London NHS Foundation Trust, London, UK; 4 Ageing and Health, Guy's and St Thomas' NHS Foundation Trust, London, UK; 5 Science and Technology Facilities Council Distributed Research Utilising Advanced Computing High Performance Computing Facility, London, UK; 6 Royal National Orthopaedic Hospital NHS Trust, Stanmore, UK; 7 Department of Computer Science, University College London, London, UK

**Keywords:** information technology

## Abstract

**Background:**

To gain maximum insight from large administrative healthcare datasets it is important to understand their data quality. Although a gold standard against which to assess criterion validity rarely exists for such datasets, internal consistency can be evaluated. We aimed to identify inconsistencies in the recording of mandatory International Statistical Classification of Diseases and Related Health Problems, tenth revision (ICD-10) codes within the Hospital Episodes Statistics dataset in England.

**Methods:**

Three exemplar medical conditions where recording is mandatory once diagnosed were chosen: autism, type II diabetes mellitus and Parkinson’s disease dementia. We identified the first occurrence of the condition ICD-10 code for a patient during the period April 2013 to March 2021 and in subsequent hospital spells. We designed and trained random forest classifiers to identify variables strongly associated with recording inconsistencies.

**Results:**

For autism, diabetes and Parkinson’s disease dementia respectively, 43.7%, 8.6% and 31.2% of subsequent spells had inconsistencies. Coding inconsistencies were highly correlated with non-coding of an underlying condition, a change in hospital trust and greater time between the spell with the first coded diagnosis and the subsequent spell. For patients with diabetes or Parkinson’s disease dementia, the code recording for spells without an overnight stay were found to have a higher rate of inconsistencies.

**Conclusions:**

Data inconsistencies are relatively common for the three conditions considered. Where these mandatory diagnoses are not recorded in administrative datasets, and where clinical decisions are made based on such data, there is potential for this to impact patient care.

**WHAT IS ALREADY KNOWN ON THIS TOPIC**

- Large-scale administrative healthcare datasets are increasingly being used to support decision-making, but very little work has been done to assess the quality and consistency of the data.

**WHAT THIS STUDY ADDS**

- The study offers a novel assessment and analysis of the data quality of the Hospital Episode Statistics dataset in the recording of mandatory diagnoses for patients with autism, type II diabetes mellitus with peripheral complications and Parkinson’s disease dementia.

**HOW THIS STUDY MIGHT AFFECT RESEARCH, PRACTICE OR POLICY**

- Data inconsistencies are relatively common for the conditions considered. Where these mandatory diagnoses are not recorded, there is potential for this to impact on the care provided. This study should motivate the improvement of clinical coding for all conditions with mandatory diagnosis recording.

## Introduction

Decision-making by clinicians and healthcare service managers is increasingly being informed by large-scale administrative healthcare data.^1^ Although such data are observational and often lack clinical details, they can support decision-making, particularly in cases where other research methods (eg, randomised controlled trial) may be considered unethical or impractical. Where such data cover an entire population of interest, they can also help minimise the risk of bias due to unrepresentative patient selection criteria (collider bias).^2^ However, it is important to have a clear understanding of data quality and the strengths and limitations of any dataset prior to analysis.^3 4^

In England, the Getting It Right First Time (GIRFT) programme is an National Health Service (NHS) England and NHS Improvement initiative with a remit to reduce unwarranted variation in clinical practice that negatively impacts on patient outcomes. The GIRFT programme is one of the largest users of administrative healthcare data for clinical outcome measurement in the UK and has a particular interest in data quality. A key data resource for the GIRFT programme is the Hospital Episodes Statistics (HES) dataset, which contains data for all hospital admissions of NHS patients in England.

The aim of this exploratory study was to identify the extent of, and data features associated with, data inconsistencies within the HES administrative dataset for England.^5^

## Methods

### Study design and data collection

This was a retrospective exploratory analysis of HES data. HES data are collected by NHS Digital for all NHS-funded patients admitted to hospitals in England. Hospital trusts run all NHS hospitals in England. A hospital trust is an administrative unit of, typically, one to four hospitals which provides secondary and/or tertiary care for all people living in a geographically defined catchment area. HES includes data for patients funded by the NHS but receiving treatment in a non-NHS hospital. Data collection and reporting is mandatory for NHS funded patients. Data are taken from clinical notes and discharge summarises and data are entered by trained clinical coders at each trust working to a national data standard.^6^ Extracts from HES data are audited against clinical audit in a small number of truss each year.

Data regarding pre-existing diagnoses would only be recorded by a coder if detailed in the medical notes or discharge summary, and all clinicians receive training in the importance of accurately recording data. Although autocoding of data is becoming more common in the NHS, its use in the period covered by our study was very limited.

HES data are primarily collected for the purposes of reimbursement. However, their value as a research resource and to inform policy decisions is being increasing recognised.^7^

In HES, a hospital spell is defined as a continuous period in hospital from admission to discharge. A spell can include multiple smaller episodes of care in various hospital settings and under different consultants. As an example, following an emergency department attendance, a patient may initially be under the care of acute medicine (episode one), then transferred to a critical care setting (episode two) and then to a care of the elderly ward (episode three) prior to discharge. Spells involving transfers to other trusts were analysed as separate spells.

### Timing, case ascertainment, inclusion and exclusion criteria

Data were taken from HES for all patient discharges during the period 1 April 2013 to 31 March 2021. Using International Statistical Classification of Diseases and Related Health Problems, tenth revision (ICD-10) codes three separate exemplar datasets were extracted for patients with a diagnosis of: childhood autism (F84.0), atypical autism (F84.1) and Asperger’s syndrome (F84.5); type II diabetes mellitus with peripheral circulatory complications (DMPC; E11.5) and Parkinson’s disease dementia (PDD; F02.3). ICD-10 codes allow data to be captured and defined consistently over time and across settings. There have been no major changes in ICD-10 coding guidance for these conditions over the study period.

DMPC and PDD were selected as representative of patients within the broader disease categories of diabetes mellitus and dementia, respectively.

These conditions were chosen for several reasons:

1. Recording of these conditions is mandated by NHS Digital and NHS England for all subsequent hospital episodes once a diagnosis has been made.^8^
2. The conditions have typical onset in childhood (autism), midlife (DMPC) and late-life (PDD) and so cover a range of demographic groups.
3. All tend to be lifelong once present, accepting that DMPC and PDD are representatives of broader conditions and that the details of the diagnosis may change within these broad definitions.

The first use of the specified code in the diagnostic record for a hospital spell during the study period was identified (index spell) and data for all subsequent spells for the same person extracted.

Spells were removed from the datasets if:

1. The only ICD-10 code present in the record was R69 (unknown and unspecified causes of morbidity) or there was no valid entry in the diagnostic code field.
2. The spell was a regular attendance for renal or liver dialysis (Office of Population Censuses and Surveys Classification of Interventions and Procedures version 4 code X40 or X43; or other regular attendance with ICD-10 code N185 (chronic renal failure) present. Regular day-attendances are usually for a specific procedure, and in most cases only that procedure and the related diagnosis is coded. Inclusion of these spells would unduly bias the dataset.
3. Patients were in age bands where the initial coding diagnoses were most likely miscoded: we removed patients with PDD younger than 40 years, and patients with DMPC younger than 18 years. The data extraction and cleaning procedure for each dataset is summarised in online supplemental figures S1–S3.

10.1136/bmjhci-2022-100633.supp1 Supplementary data

### Identification of data inconsistencies

All data inconsistencies are reported at the spell level. A subsequent spell was considered consistent with the first spell if at least one of its constituent episodes mentioned the ICD-10 codes listed below for that condition:

Autism: F84.0, F84.1 or F84.5.

DMPC (representing the broader disease category of diabetes mellitus): E10-, E11-, E14-.

PDD (representing the broader disease category of dementia): F00-, F01-, F02-, F03-, F05.1, G30.1, G30.8, G309.

Further details on the definitions of these codes are summarised in online supplemental table S1. In the case of DMPC and PDD, a broader definition of the condition was used for subsequent spells than for the first spell. This was in recognition of the fact that details may not be recorded regarding the diabetes subtype or its presentation or the exact role of Parkinson’s disease in the development of dementia.

### Covariates and data features/characteristics

Patient characteristics: sex, age in years, ethnicity (white, black or black British, Asian or Asian British, mixed, other and not stated), comorbidities (Charlson Comorbidity Index,^9^ frailty (Hospital Frailty Risk Score (HFRS)^10^ and the Global Frailty Score,^11^ and deprivation (Index of Multiple Deprivation scores).^12^

Features of hospital stay: Spell length of stay, admission method (emergency or elective), main specialty, number of days since the first spell with the diagnosis recorded (reported as the difference between the discharge date of the first spell and the admission date of the subsequent spell), change of trust between the first and subsequent spell, change of clinical specialty between the first and subsequent spell.

Coding of underlying conditions: We identified spells where a related condition would be expected to also be diagnosed. For PDD this was Parkinson’s disease (ICD-10 code G20), and for autism, whether learning disability (ICD-10 codes F70-, F71-, F72-, F73-, F78-, F79-, F80-, F81-, F82- or F83-) was also mentioned in the diagnostic record. The Parkinson’s disease code is not mandatory, although the learning disability codes are mandatory.

### Outcome (target) variable

For each condition, the target was described by a binary flag indicating whether a code was recorded in the subsequent spell.

### Data analysis

Data were extracted onto a secure encrypted server controlled by NHS England and NHS Improvement. Analysis within this secure environment took place using Alteryx 2019.3 (Alteryx, Irvine, California, USA), Python V.3.9.6 and the scikit-learn machine learning library V.1.0.1 (Python Software Foundation, Beaverton, Oregon, USA).^13^

Important predictors associated with data inconsistencies were identified using a random forest classifier algorithm (briefly described in online supplemental figure S4). Missing data values were handled by imputation with the mean or mode in each class. The datasets were separated into a training, validation and test sets with 70%, 15% and 15% of data respectively. Machine learning algorithms require the data to be randomly split so that the algorithm can learn the relationships between the data points and then apply this learning to an unseen part of the data set. The algorithm parameters were determined using the validation set by performing a randomised search on a grid of values and choosing the ones that led to the highest value for the area under the precision recall curve. The classifiers were then trained on the training set and evaluated on the withheld test set. The final parameters of each classifier are summarised in online supplemental table S2.

The models’ most important predictors were identified using the SHapley Additive exPlanation (SHAP) feature importance^14^ to minimise bias towards high-cardinality variables. Positive or negative correlations of predictors with coding inconsistencies were estimated by calculating the Kendall Tau-b correlation coefficients between the values of the variables, and their estimated Shapley values. These were calculated using TreeSHAP, an efficient estimation approach for tree-based models.^15^ Model performance was evaluated using the area under the receiver operating characteristics (AUROC) curve, precision-recall curves and precision gain—recall gain curves.^16^ CIs for the areas under the curves were computed using a python implementation of the DeLong method.^17 18^

In subanalyses, we evaluated the impact of time from the first spell on the proportion of inconsistencies. Time from admission for the first spell where the diagnostic code was used to admission for a subsequent spell was calculated in days for the subset of patients where the first spell was prior to 1 April 2018. The follow-up period was set at 3 years for all patients. This was done to avoid a potential bias due to varying maximum follow-up periods for each patient.

## Results

Data were available for 172 324 unique patients with autism, 106 943 unique patients with DMPC and 27 794 unique patients with PDD. The characteristics of these patients on their first spell during the study period are summarised in table 1 together with the number of patients without data recorded for each feature. Autism patients had the youngest and patients with PDD the oldest age structure. The autism and DMPC dataset had a high proportion of patients from more deprived areas.

**Table 1:** Table of patient characteristics on first spell within the study period

	Autism	Diabetes mellitus with peripheral complications	Parkinson’s disease dementia
No of patients	172 324	106 943	27 794
Age band			
0–17	98 591 (57.2 %)	8 (0.01 %)	0 (0.0 %)
18–39	50 682 (29.4 %)	1085 (1.0 %)	12 (0.04 %)
40–59	16 060 (9.3 %)	21 745 (20.3 %)	279 (1.0%)
60–79	6171 (3.6 %)	55 050 (51.5 %)	12 375 (44.5 %)
80 years and over	820 (0.5 %)	28 938 (27.1 %)	15 111 (54.4 %)
Not recorded	0	117	17
Sex			
Female	49 414 (28.7 %)	32 854 (30.7 %)	9828 (35.4 %)
Male	122 616 (71.2 %)	74 089 (69.3 %)	17 961 (64.6 %)
Not recorded	294	0	5
Deprivation quintile			
1 (most deprived)	48 539 (29.1 %)	27 136 (25.4 %)	4475 (16.1 %)
2	38 254 (22.9 %)	23 419 (21.9 %)	5248 (18.9 %)
3	31 311 (18.28%)	20 714 (19.4 %)	5815 (20.9 %)
4	26 332 (15.8 %)	17 008 (15.9 %)	6084 (21.9 %)
5 (least deprived)	22 275 (13.4 %)	13 757 (12.9 %)	5790 (20.8 %)
Not recorded	5613	4909	382
Ethnicity			
White	113 146 (77.9 %)	89 084 (84.8 %)	21 402 (93.6 %)
Asian	6916 (4.8 %)	4778 (4.5 %)	730 (3.2 %)
Black	4964 (3.4 %)	3371 (3.2 %)	426 (1.9 %)
Mixed	3695 (2.5 %)	435 (0.4 %)	61 (0.3 %)
Other ethnic groups	16 537 (11.4%)	7325 (7.0 %)	240 (1.1 %)
Not recorded	27 066	1950	4935
Most common specialties	Paediatrics (23.5 %)	General medicine (33.4 %)	General medicine (33.1%)
	General surgery (7.3 %)	General surgery (31.9 %)	Geriatrics medicine (20.7 %)

Where data are not recorded for deprivation, this is due to the lower super output area of residence not being recorded. In most cases this is due to the patient not having a permanent residence in England (typically they be residents of other parts of the UK). Percentages for each recorded category are calculated excluding any unrecorded data.

**Figure 1:**
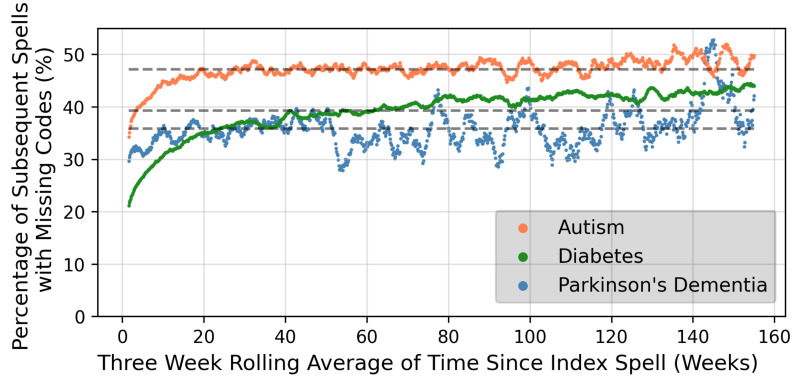
Proportion of subsequent spells with inconsistencies over time up to three years after the index spell

**Figure 2:**
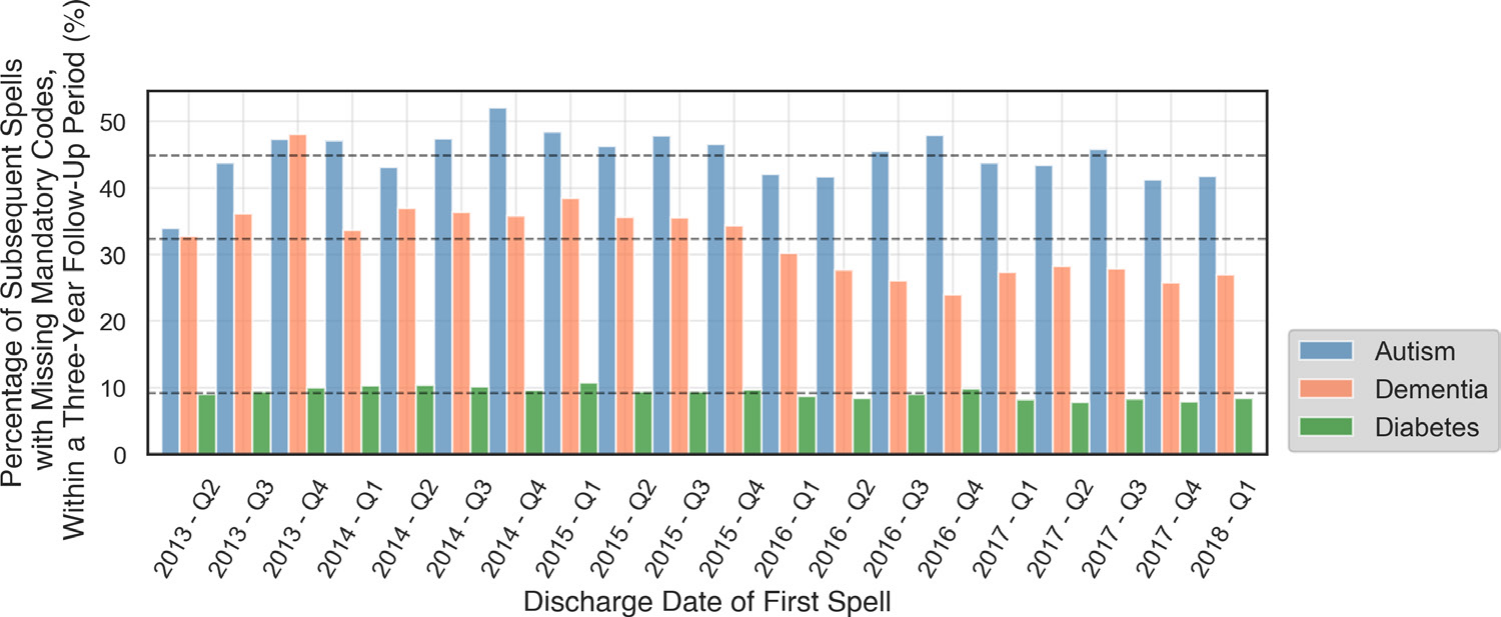
Percentage of spells with missing mandatory codes within 3 years of the first diagnosis, for the discharge date of the first spell ranging from Q2-2013 to Q1-2018.

The number of subsequent spells for each patient within a 3-year follow-up period are shown in online supplemental figure S5 for each condition. High numbers of patients (more than 50% for patients with autism) had no subsequent spells within 3 years of their first spell. Patients with DMPC had the highest numbers of subsequent spells. Figure 1 summarises the number of data inconsistencies in these subsequent spells up to 3 years from the first spell where the diagnostic code was used. The number of data inconsistencies increased with time from the first spell, although the trend was less obvious after approximately 20 weeks. Figure 2 illustrate the percentage of subsequent spells with missing mandatory codes in the 3 years after the first spell. The consistency of the coding for PDD appeared to broadly improve over the study period, while for autism patients, consistency appears to have decreased slightly over time.

The number of subsequent spells with data inconsistencies were 170 447 (43.7%) for patients with autism, 46 679 (8.6%) for DMPC patients and 18 975 (31.2%) for patients with PDD. The number of subsequent spells with inconsistencies according to patient characteristics is summarised in table 2. For people with autism, data inconsistencies became more common with greater age. However, for PDD inconsistencies became less common with greater age. Females with autism and PDD had a noticeably higher proportion of inconsistencies than males. There was a modest trend towards a higher proportion of data inconsistencies in autism patients with increasing deprivation. White patients had the highest rate of inconsistencies for autism.

**Table 2:** Characteristics of subsequent spells with data inconsistencies

	Autism	Diabetes mellitus with peripheral complications	Parkinson’s disease dementia
Total no of spells	583 873	651 458	91 328
No of subsequent spells	390 220	544 341	60 822
No of subsequent spells with missing mandatory codes	170 447 (43.7 %)	46 679 (8.6 %)	18 975 (31.2 %)
Data inconsistencies by overnight stays			
Overnight stay	66 251 (38.9 %)	16 792 (5.0 %)	11 134 (25.2 %)
Day case	104 196 (47.3 %)	29 887 (14.2 %)	7841 (46.8 %)
Data inconsistencies by method of admission			
Elective	84 845 (44.4 %)	19 393 (5.6 %)	6214 (57.8 %)
Emergency	85 380 (43.0 %)	27 247 (13.9 %)	12 752 (25.5 %)
Not recorded	222 (42.4 %)	39 (24.7 %)	9 (32.1 %)
Data inconsistencies by age band			
0–17	57 317 (35.2 %)	0	0
18–39	71 936 (47.3 %)	594 (9.9 %)	0
40–59	27 441 (54.4 %)	9065 (7.3 %)	502 (60.5 %)
60–79	12 025 (53.7 %)	25 409 (8.6 %)	9514 (34.0 %)
80 years and over	1728 (65.2 %)	11 597 (9.7 %)	8955 (28.0 %)
Not recorded	0	14 (6.6 %)	7 (23.5 %)
Data inconsistencies by sex			
Female	64 650 (46.7 %)	14 150 (8.8 %)	6554 (32.9 %)
Male	105 797 (42.0 %)	32 529 (8.5 %)	12 421 (30.4 %)
Not recorded/other	0	0	0
Data inconsistencies by deprivation quintile			
1 (most deprived)	50 739 (45.1 %)	11 298 (7.3 %)	3466 (29.2 %)
2	40 305 (44.8 %)	10 383 (8.1 %)	3622 (30.0 %)
3	30 392 (41.5 %)	9493 (8.8 %)	5000 (36.1 %)
4	26 390 (43.0 %)	8428 (9.9 %)	3587 (30.0 %)
5 (least deprived)	20 478 (41.8 %)	6811 (10.1 %)	3213 (29.9 %)
Not recorded	2143 (50.1 %)	266 (9.7 %)	87 (30.5 %)
Data inconsistencies by ethnicity			
White	114 938 (42.9 %)	39 129 (8.5 %)	14 301 (31.4 %)
Asian	4733 (36.3 %)	1811 (7.0 %)	720 (35.1 %)
Black	4195 (41.3 %)	1301 (7.5 %)	295 (25.5 %)
Mixed	2362 (36.3 %)	385 (14.8 %)	48 (34.0 %)
Other ethnic groups	10 155 (41.8 %)	2968 (9.8 %)	183 (32.6 %)
Not recorded/stated	34 064 (42.9 %)	1086 (12.8 %)	3428 (30.0 %)

Where data are not recorded for deprivation, this is due to the lower super output area of residence not being recorded. In most cases this is due to the patient not having a permanent residence in England (typically they would be residents of other parts of the UK).

The variation in data inconsistencies across trusts in England is summarised in online supplemental figure S6. There was substantial spread in terms of data inconsistencies across trusts.

**Figure 3:**
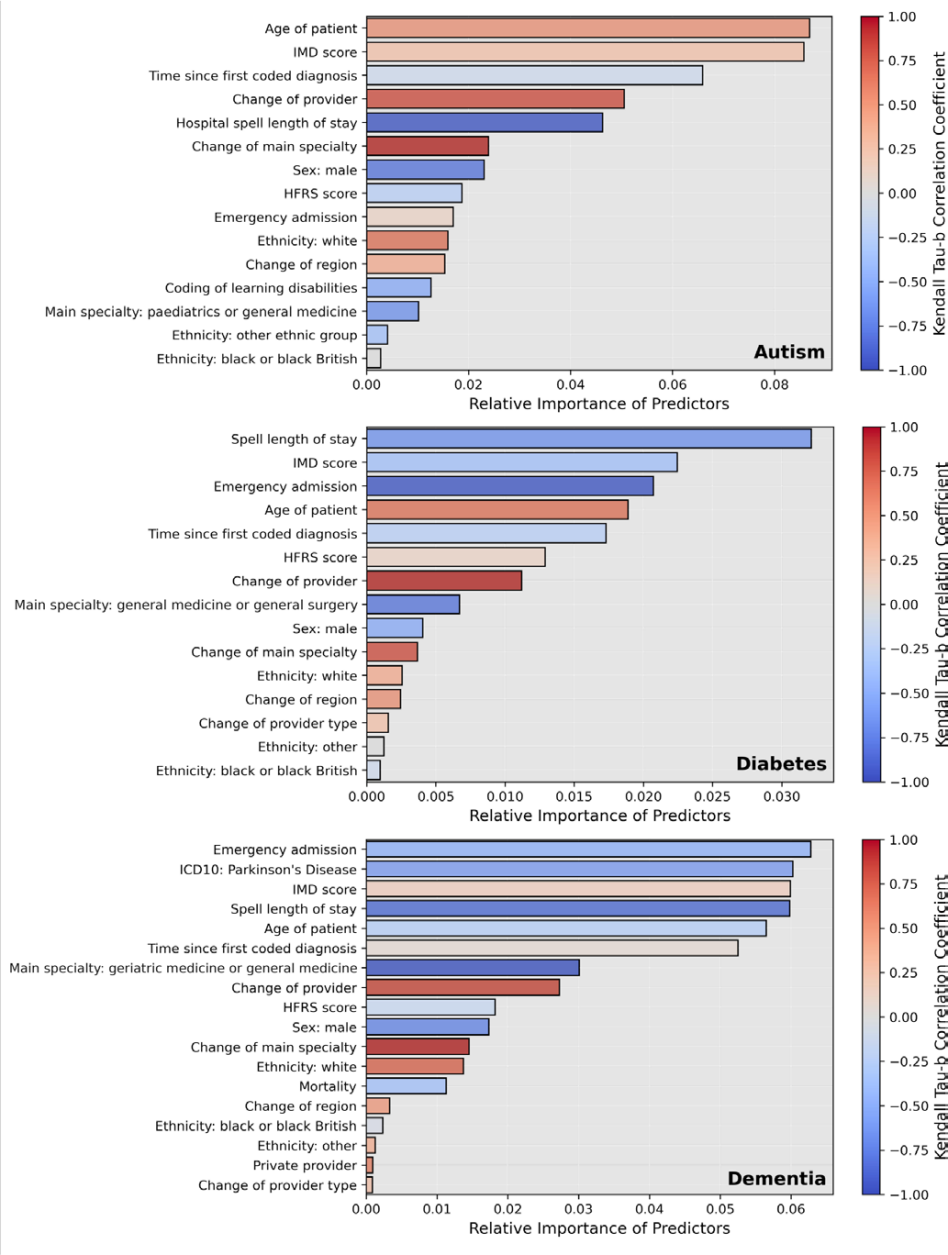
Relative permutation importance of predictors contributing to the identification of coding inconsistencies at the spell level for diagnoses of autism (top), diabetes mellitus with peripheral complications (middle) and Parkinson's disease dementia (bottom). Note: The length of each bar indicates how strongly the classifiers rely on each variable to predict coding consistency at the spell level in the test sets; it is a measure of the relative importance of each predictor. The colour bars indicate the values of the Kendall tau-b correlation coefficient between the values of each variable and the estimated Shapley values. Coefficients close to 1 or -1 correspond to strong positive or negative correlations with coding inconsistencies respectively. HFRS, Hospital Frailty Risk Score; ICD-10, International Statistical Classification of Diseases and Related Health Problems, 10th revision; IMD, Index of Multiple Deprivation.

Three random forest classifiers were optimised and trained to identify coding inconsistencies for each condition. The relative importance of each feature is shown in figure 3. Across all three conditions, features strongly associated with data inconsistencies included a change in specialty, a change in provider, shorter spell length of stay and female sex. Data inconsistencies were also associated with older patient age for autism and DMPC and younger patients age for PDD. Although deprivation score was an important predictor for all three conditions, the directionality of the relationship was unclear. For patients with PDD, emergency admissions and the absence of the diagnostic code for Parkinson’s disease were the most important features. AUROC curve values were 0.80 (95% CI 0.80 to 0.81) for autism, 0.76 (95% CI 0.76 to 0.77) for DMPC and 0.75 (95% CI 0.73 to 0.76) for PDD. online supplemental figure S7 reports the areas under the precision-recall curves and precision gain—recall gain curves, also suggesting the classifiers to have good performance. The performance of each model in Black, Asian, male and female patient subgroups is summarised in Online supplemental table S3o and indicates no significant drop in performance for these groups.

## Discussion

We used machine learning algorithms to analyse three large datasets to investigate the consistency of clinical coding of three mandatory health conditions within a large administrative healthcare dataset. Clinical coding of DMPC as a mandatory condition was relatively consistent. However, over two-fifths of subsequent spells for autism patients and almost a quarter of subsequent spells for patients with PDD had data inconsistencies. There was a high level of variation in the proportion of data inconsistencies between trusts, and there was no evidence that trusts are consistently poor at reporting mandatory codes across the three conditions studied.

In the HES dataset, inconsistencies related to mandatory clinical codes can arise from two main sources. A failure of the clinician to record the diagnosis in the medical notes or a failure of the clinical coder to code a diagnosis recorded in medical notes. In our analysis, data inconsistencies could also be due to misuse of the code of interest on the first spell (ie, a false positive in the index spell), although the numbers involved are likely to be small.

From the random forest classifier algorithms, age was strongly associated with data inconsistencies. A greater proportion of data inconsistencies were associated with increasing age for autism and DMPC, and with decreasing age for PDD. This confirms the pattern seen in the descriptive data and is likely to be due to expectations around the likelihood that a patient has the condition. This may also explain the relative importance of the association between female sex and more inconsistencies in the autism dataset. Although we identified a relationship between deprivation score and data inconsistencies in all three datasets, the nature of the relationship was unclear. This may suggest a bias towards continuous variables in the algorithms used.^19 20^

Change in provider, change in main specialty and time from first spell to the subsequent admission were also associated with a higher proportion of data inconsistencies across all datasets. Initiatives to allow easier cross-referencing of information across providers and settings and over an extended period of time should be encouraged.

For the PDD dataset, coding of Parkinson’s disease and emergency admission were associated with lower rates of inconsistencies. Elective admissions are generally of short duration and the case notes are likely to focus on the elective procedure being conducted, with limited coding depth.

Large scale, administrative datasets, such as HES, are being increasingly used to inform decision-making in healthcare.^21 22^ Such data have helped inform the response to the COVID-19 pandemic^23 24^ and are being used to inform service structure postpandemic.^25–27^ Having data which is as reliable as possible will be invaluable. Understanding the source and structure of coding inconsistencies may also help the development of new quality improvement programmes, as well as inform the work of researchers, clinical coders and policy analysts.^22 28^ The impact of the data inconsistencies identified in this paper will vary in importance depending on the nature and aims of the data analysis being undertaken. However, we recommend that researchers using HES and interested in long-term comorbidities should not rely on the coding of the index spell alone, but should look at prior spells for the same patient. Frailty/comorbidity indices, such as the Charlson Comorbidity Index and HFRS, if constructed from HES data, perform this function (to an extent) by looking back over 1 and 2 years of prior hospital spells, respectively.

The performance of the algorithms used to identify key features of data inconsistencies was similar in smaller subgroups of ethnicity and sex. There are concerns that artificial intelligence (AI) techniques can accentuate known biases against representation of smaller subpopulations of a dataset.^29 30^ Although the problem of fair data analysis is not unique to AI techniques, and can occur with more traditional forms of data processing and analysis, the ‘black-box’ element of AI methodology leads naturally to concerns over ‘fair AI’ and data equity. We used random forest classifiers in our analysis, allowing us to understand the key features represented in our algorithms and allowing a degree of transparency.

Our study has a number of strengths and limitations. We had access to one of the most extensive and complete healthcare datasets anywhere in the world. However, this meant that there was no ‘gold standard’ against which to externally validate the dataset. Difference in coding practice across trusts will have affected our assessment of data quality on the national scale, and we highlight the variation across trusts. We were not able to identify whether an inconsistency was related to a mandatory code being misused in a first spell or being missing in all subsequent spells. We recognise that patients with diabetes mellitus can go into remission, but the number involved across the time period investigated are likely to be very small indeed. We also acknowledge that some forms of dementia and autism may be mild and not impact on the clinical care. Nevertheless, all the conditions studied are mandatory and should still be recorded once diagnosed. Given the potential variability in the source and proportions of coding inconsistencies across all three conditions, the performance of the three classifiers should not be assessed by one single metric alone. For that reason, we opted to also use the precision-recall curves and the recall-aware precision gain—recall gain curves, particularly relevant for the coding of diabetes where the number of inconsistencies is much lower (ie, higher class imbalance). Our analysis highlights that the characteristics of coding inconsistencies can be particular to the condition under investigation. Although we selected conditions that tend to be present across the lifetime, extrapolation to other disease groups should be done with caution. More broadly, although we investigated inconsistent use of mandatory diagnostic codes in this study, it would be possible to investigate other types of inconsistences using similar methods.

## Conclusions

We have identified the extent of, and features associated with, data inconsistencies in the HES database for the three conditions studied, with autism having the highest rate of data inconsistencies. With the likely increased use of administrative data to inform healthcare decision-making, data quality will be of central importance if outcomes for patients are to be optimised. As such, improving data quality should be a priority.

Machine learning techniques, as well as providing insight into the characteristics associated with data inconsistencies, may also be of value in identifying potential data inconsistencies during data input, allowing inconsistencies to be corrected prior to finalisation of the data submission.

## Data Availability

No data are available. Requests for any underlying data cannot be granted by the authors because the data were acquired from data under licence/data sharing agreement from NHS Digital, for which conditions of use (and further use) apply. Individuals and organisations wishing to access HES data can make a request directly to NHS Digital.

## References

[1] Agrawal R, Prabakaran S. Big data in digital healthcare: lessons learnt and recommendations for general practice. Heredity 2020;124:525–34. 10.1038/s41437-020-0303-2 32139886 PMC7080757

[2] Griffith GJ, Morris TT, Tudball MJ, et al. Collider bias undermines our understanding of COVID-19 disease risk and severity. Nat Commun 2020;11:1–12. 10.1038/s41467-020-19478-2 33184277 PMC7665028

[3] Stulberg JJ, Haut ER. Practical guide to surgical data sets: healthcare cost and utilization project national inpatient sample (NIS). JAMA Surg 2018;153:586–7. 10.1001/jamasurg.2018.0542 29617533

[4] Benchimol EI, Smeeth L, Guttmann A, et al. The reporting of studies conducted using observational Routinely-collected health data (record) statement. PLoS Med 2015;12:e1001885. 10.1371/journal.pmed.1001885 26440803 PMC4595218

[5] Oswald M. Anonymisation standard for publishing health and social care data specification (process standard. Leeds, UK: Information Standards Board for Health and Social Care, 2013.

[6] NHS Digital. National clinical coding standards ICD-10Accurate data for quality information. 5th Edition. Leeds, UK: Terminology and Classifications Delivery Service, 2021.

[7] Herbert A, Wijlaars L, Zylbersztejn A, et al. Data resource profile: Hospital episode statistics admitted patient care (Hes APC). Int J Epidemiol 2017;46:1093–1093i. 10.1093/ije/dyx015 28338941 PMC5837677

[8] NHS Digital. National clinical coding standards ICD-10. 2021. 5th edn, 2021. https://classbrowser.nhs.uk/ref_books/ICD-10_2021_5th_Ed_NCCS.pdf

[9] Sundararajan V, Henderson T, Perry C, et al. New ICD-10 version of the Charlson comorbidity index predicted in-hospital mortality. J Clin Epidemiol 2004;57:1288–94. 10.1016/j.jclinepi.2004.03.012 15617955

[10] Gilbert T, Neuburger J, Kraindler J, et al. Development and validation of a hospital frailty risk score focusing on older people in acute care settings using electronic Hospital records: an observational study. Lancet 2018;391:1775–82. 10.1016/S0140-6736(18)30668-8 29706364 PMC5946808

[11] Soong JTY, Kaubryte J, Liew D, et al. Dr foster global frailty score: an international retrospective observational study developing and validating a risk prediction model for hospitalised older persons from administrative data sets. BMJ Open 2019;9:e026759. 10.1136/bmjopen-2018-026759 PMC6596946 31230009

[12] Ministry of Housing and Communities and Local Government. English indices of deprivation, 2019. Available: https://www.gov.uk/government/collections/english-indices-of-deprivation [Accessed 25 Aug 2021].

[13] Pedregosa F, Varoquaux G, Gramfort A, et al. Scikit-learn: machine learning in python. Journal of machine Learning research 2011;12:2825–30.

[14] Lundberg SM, Lee S-I. A unified approach to interpreting model predictions. Proceedings of the 31st international conference on neural information processing systems 2017;2017:4768–77.

[15] Lundberg SM, Erion GG, Lee S-I. Consistent individualized feature Attribution for tree ensembles. arXiv preprint arXiv 2018:180203888.

[16] Flach P, Kull M. Precision-recall-gain curves: PR analysis done right. Adv Neural Inf Process Syst 2015;28.

[17] DeLong ER, DeLong DM, Clarke-Pearson DL. Comparing the areas under two or more correlated receiver operating characteristic curves: a nonparametric approach. Biometrics 1988;44:837–45. 10.2307/2531595 3203132

[18] Sun X, Xu W. Fast implementation of DeLong’s algorithm for comparing the areas under correlated receiver operating characteristic curves. IEEE Signal Process Lett 2014;21:1389–93. 10.1109/LSP.2014.2337313

[19] Strobl C, Boulesteix A-L, Zeileis A, et al. Bias in random forest variable importance measures: illustrations, sources and a solution. BMC Bioinformatics 2007;8:1–21. 10.1186/1471-2105-8-25 17254353 PMC1796903

[20] Strobl C, Boulesteix A-L, Kneib T, et al. Conditional variable importance for random forests. BMC Bioinformatics 2008;9:1–11. 10.1186/1471-2105-9-307 18620558 PMC2491635

[21] Gray WK, Day J, Horrocks M. Editor's Choice - Volume-Outcome Relationships in Elective Abdominal Aortic Aneurysm Surgery: Analysis of the UK Hospital Episodes Statistics Database for the Getting It Right First Time (GIRFT) Programme. Eur J Vasc Endovasc Surg 2020;60:509–17. 10.1016/j.ejvs.2020.07.069 32807679

[22] Nouraei SAR, Mace AD, Middleton SE, et al. A stratified analysis of the perioperative outcome of 17623 patients undergoing major head and neck cancer surgery in England over 10 years: towards an Informatics-based outcomes surveillance framework. Clin Otolaryngol 2017;42:11–28. 10.1111/coa.12649 26990866

[23] Navaratnam AV, Gray WK, Day J, et al. Patient factors and temporal trends associated with COVID-19 in-hospital mortality in England: an observational study using administrative data. Lancet Respir Med 2021;9:397–406. 10.1016/S2213-2600(20)30579-8 33600777 PMC7906650

[24] Gray WK, Navaratnam AV, Day J, et al. Changes in COVID-19 in-hospital mortality in hospitalised adults in England over the first seven months of the pandemic: an observational study using administrative data. Lancet Reg Health Eur 2021;5:100104. 10.1016/j.lanepe.2021.100104 33969337 PMC8086562

[25] Model Health System. Model health system: supporting NHS teams to provide high quality patient care and continuous improvement, 2022. Available: https://model.nhs.uk/ [Accessed 02 Jul 2022].

[26] National Clinical Improvement Programme. National clinical improvement programme, 2022. Available: https://www.gettingitrightfirsttime.co.uk/associated-projects/ncip/ [Accessed 02 Jul 2022].

[27] Gray WK, Takhar AS, Navaratnam AV, et al. Safety of day-case paediatric tonsillectomy in England: an analysis of administrative data for the getting it right first time programme. Anaesthesia 2022;77:277–85. 10.1111/anae.15562 34530496

[28] Jewell A, Broadbent M, Hayes RD, et al. Impact of matching error on linked mortality outcome in a data linkage of secondary mental health data with Hospital episode statistics (Hes) and mortality records in South East London: a cross-sectional study. BMJ Open 2020;10:e035884. 10.1136/bmjopen-2019-035884 PMC7342822 32641360

[29] Reddy S, Fox J, Purohit MP. Artificial intelligence-enabled healthcare delivery. J R Soc Med 2019;112:22–8. 10.1177/0141076818815510 30507284 PMC6348559

[30] Panch T, Mattie H, Atun R. Artificial intelligence and algorithmic bias: implications for health systems. J Glob Health 2019;9:010318. 10.7189/jogh.09.020318 PMC6875681 31788229

