# Supplementary material for: Data consistency in the English Hospital Episodes Statistics database
Source: BMJ Health Care Inform. 2022 Oct 28;29(1):e100633. doi: 10.1136/bmjhci-2022-100633 (PMC9621173; doi:10.1136/bmjhci-2022-100633)

Supplementary Table S1: Table of patient characteristics

Condition	ICD-10 codes used to identify the condition	ICD-10 codes used to identity data consistencies subsequent spells
**Parkinson's disease dementia**	**F02.3**: Dementia in Parkinson disease	**F00**: Dementia in Alzheimer disease<br>**F01**: Vascular dementia<br>**F02**: Dementia in other diseases, classified elsewhere<br>**F03**: Unspecified dementia<br>**F05.1**: Delirium superimposed on dementia<br>**G30.0**: Alzheimer disease with early onset<br>**G30.1**: Alzheimer disease with late onset<br>**G30.8**: Other Alzheimer disease<br>or **G30.9**: Alzheimer disease, unspecified
**Autism**	**F84**: Autism	**F84.0**: Childhood autism<br>**F84.1**: Atypical autism<br>or **F84.5**: Asperger syndrome
**Type 2 Diabetes Mellitus**	**E11**: Type 2 diabetes mellitus	**E10**: Type 1 diabetes mellitus<br>**E11**: Type 2 diabetes mellitus<br>or **E14**: Unspecified diabetes mellitus

Hardy F, *et al. BMJ Health Care Inform* 2022; 29:e100633. doi: 10.1136/bmjhci-2022-100633

Supplementary Table S2: Parameters and performance of optimised Random Forest classifiers

	**Autism**	**Diabetes mellitus with peripheral complications**	**Parkinson's disease dementia**
**AUROC curve (95% confidence interval)**	0.80 [0.80-0.81]	0.76 [0.76-0.77]	0.75 [0.73-0.76]
**Parameters**			
n_estimators	242	136	242
min_samples_split	6	20	6
min_samples_leaf	1	1	1
max_features	sqrt	auto	sqrt
max_depth	93	62	93
class_weight	balanced	None	None
bootstrap	False	False	balanced

AUROC = area under the receiver operating characteristic

Supplementary Table S3: Performance of models on sub-groups of patients

	**Autism**		**Diabetes mellitus with peripheral complications**		**Parkinson's disease dementia**	
	Percentage of population	AUROC [95% CI]	Percentage of population	AUROC [95% CI]	Percentage of population	AUROC [95% CI]
**Ethnicity**						
Black	2.6 %	0.88 [0.87-0.90]	3.3 %	0.80 [0.76-0.83]	2.2 %	0.75 [0.66-0.84]
Asian	5.0 %	0.81 [0.81-0.82]	4.8 %	0.78 [0.76-0.81]	3.8 %	0.77 [0.75-0.80]
**Sex**						
Female	35.3 %	0.78 [0.77-0.78]	30.0 %	0.76 [0.75-0.78]	33.4%	0.75 [0.72-0.76]
Male	64.7 %	0.82 [0.81-0.82]	70.4 %	0.76 [0.75-0.76]	66.6%	0.74 [0.73-0.76]

AUROC = area under the receiver operating characteristic

Supplementary Figure S1: Flow diagrams of pruning procedures for the identification of coding inconsistencies in patients with autism

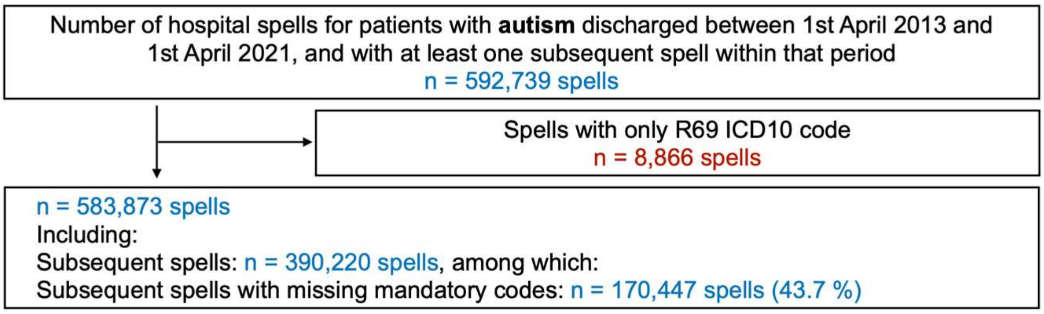

Hardy F, *et al. BMJ Health Care Inform* 2022; 29:e100633. doi: 10.1136/bmjhci-2022-100633

Supplementary Figure S2: Flow diagrams of pruning procedures for the identification of coding inconsistencies in patients with diabetes mellitus with peripheral complications

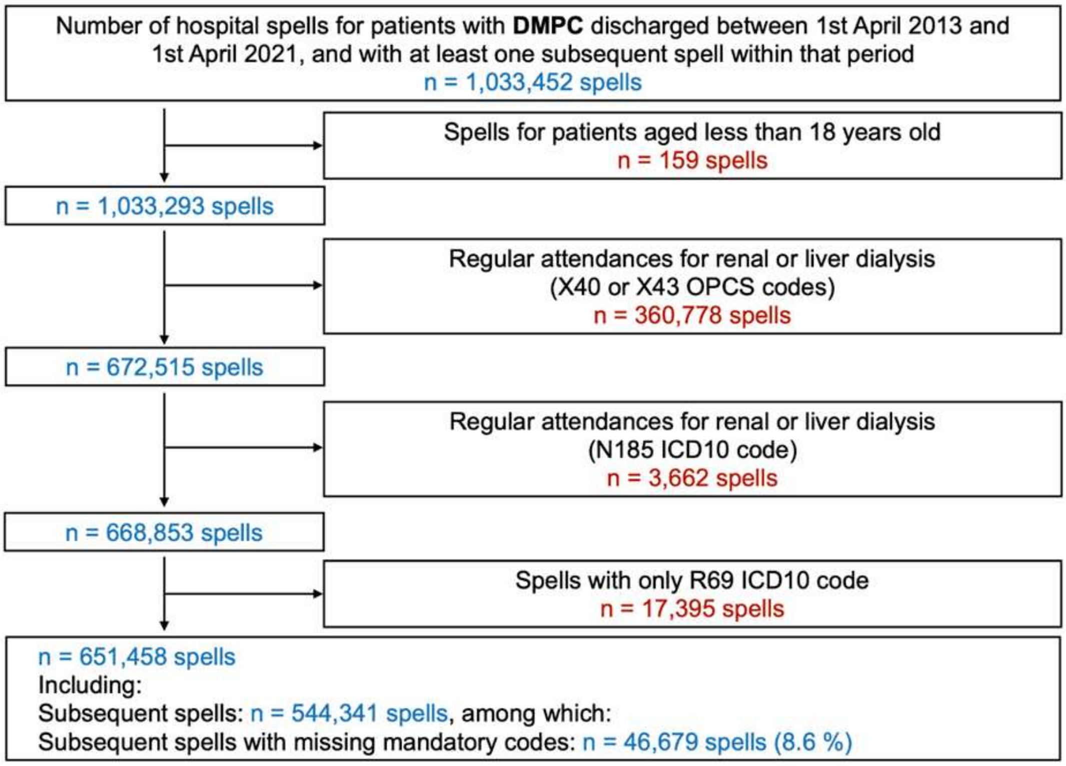

Hardy F, *et al. BMJ Health Care Inform* 2022; 29:e100633. doi: 10.1136/bmjhci-2022-100633

Supplementary Figure S3: Flow diagrams of pruning procedures for the identification of coding

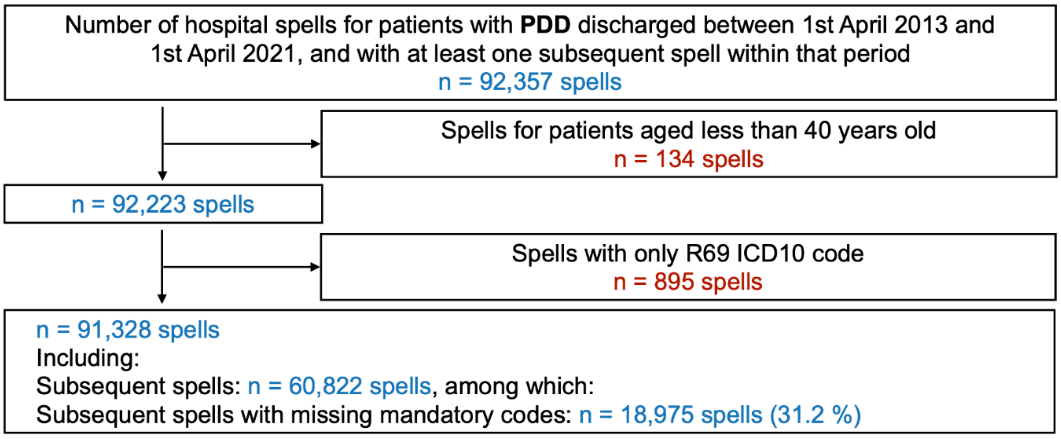

inconsistencies in patients with Parkinson's disease dementia

Supplementary material Figure S4: Description of a random forest classifier
A random forest is an ensembled-based method which comprises the construction of many simple decision trees in the training stage, before making a prediction using the majority vote across all trees.

The collection trees are built on a set of bootstrap samples generated from the original training set. The nodes of each tree are split according to a measure (often the *Gini impurity*) associated with a selected subset of variables.

Once the decision trees are built, a prediction is made from a new datapoint (e.g. from the test set) using a majority vote across all trees.

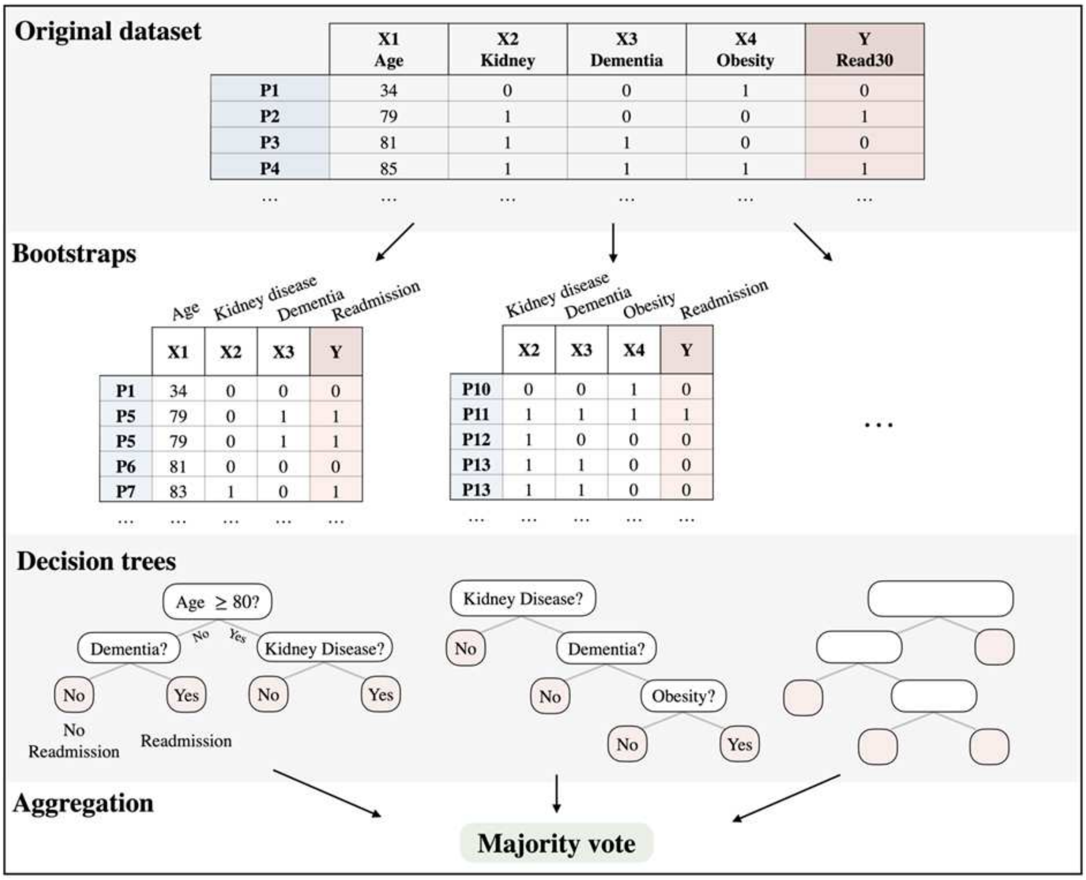

Supplementary material Figure S5: Number of subsequent spells per patient within a three-year follow-up period for each condition.

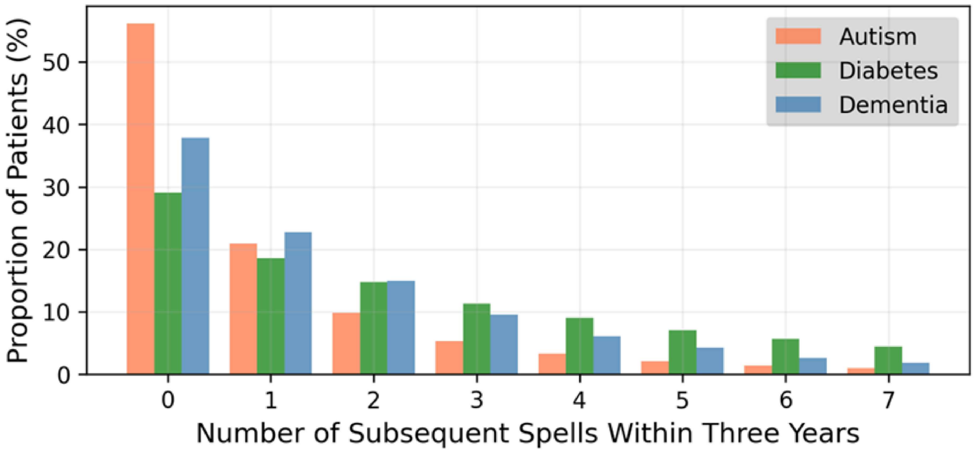

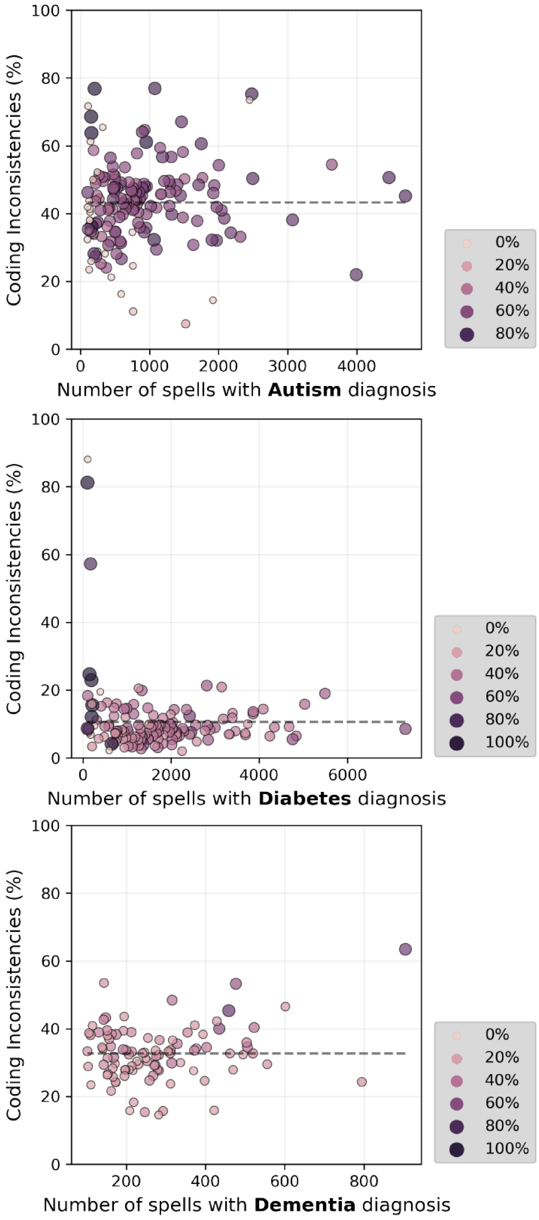

Supplementary material Figure S6: Proportion of spells with data inconsistencies across trusts.

Proportion of coding inconsistencies across trusts, as a function of numbers of recorded spells. The marker size and colour-scale correspond to the proportion of spells with zero-day length of stay.

Supplementary Figure S7: Performance curves of all random forest classifiers for coding inconsistencies related to diagnoses of autism (top), diabetes mellitus with peripheral complications (middle) and Parkinson's disease dementia (bottom).

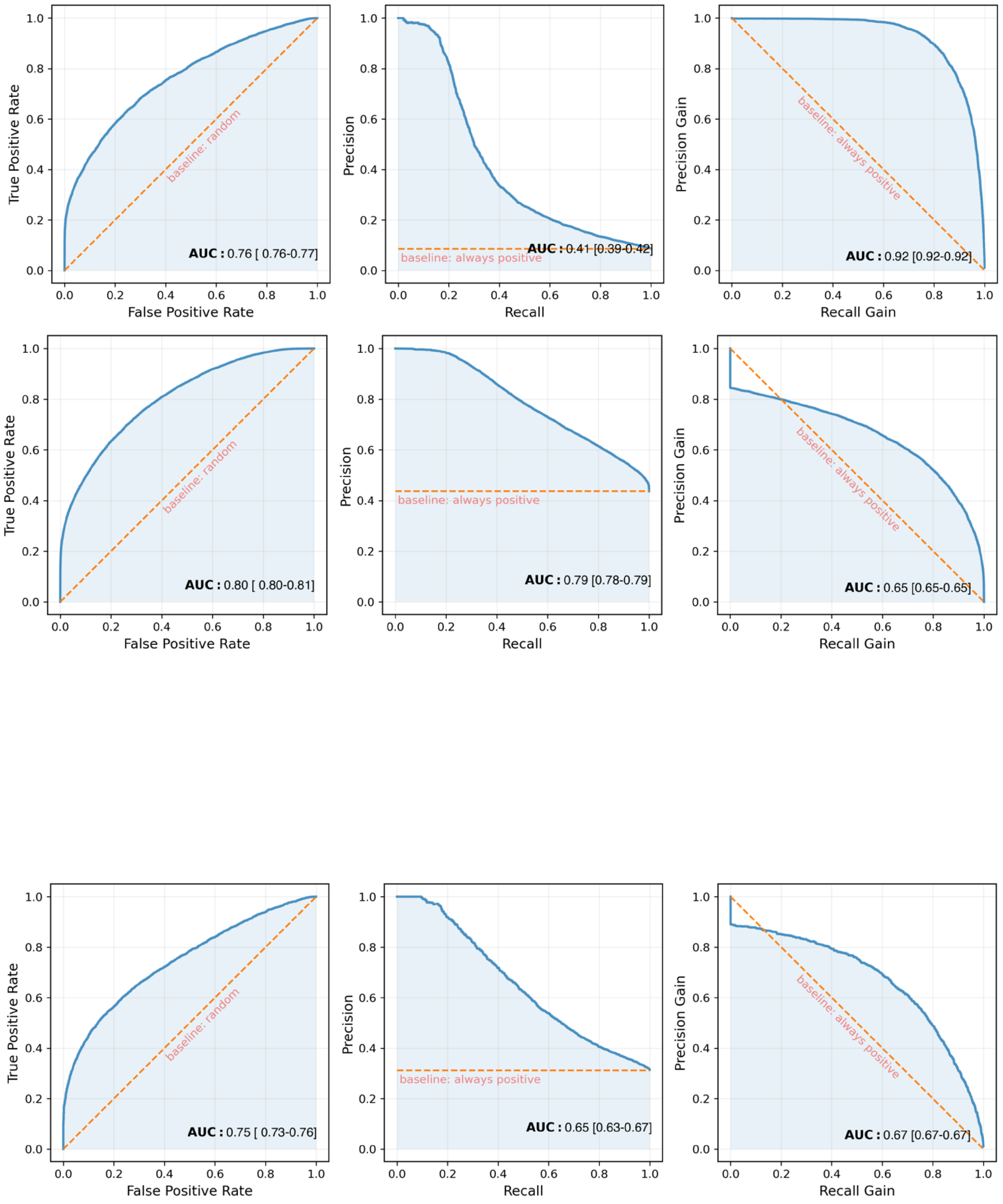

Supplement: Supplementary data [file bmjhci-2022-100633supp001.pdf]
